# Supplementary material for: How does analyst coverage influence corporate social responsibility (CSR)? The governance- and information-based perspectives
Source: PLoS One. 2024 Apr 29;19(4):e0302165. doi: 10.1371/journal.pone.0302165 (PMC11057776; doi:10.1371/journal.pone.0302165)
Supplement: Attachment — Submitted filename: Response letter.docx [file pone.0302165.s002.docx]

Response Letter

It is with excitement that we resubmit to you a revised version of manuscript “**How does analyst coverage influence corporate social responsibility (CSR)? The governance- and information-based mechanisms (PONE-D-23-17772)**” for the ***Plos One***. Many thanks to all the editors for their valuable time, constructive comments, and valuable suggestions. Based on the review comments, we completed the revision of the manuscript with the full cooperation of the author team and resubmitted the new version before the deadline. We thank you for your time and details provided. We have incorporated the suggested revisions into this refined manuscript to the best of our ability. We have benefited greatly from these insightful suggested revisions. We look forward to working with you to bring the manuscript closer to publication in ***Plos One***.

Based on the editor and reviewers’ comments and suggestions, we have carefully revised the manuscript. The following is a detailed response to the editor and reviewers’ comments.

*Reviewer #1: This paper documents the positive association between analyst coverage and CSR. Authors demonstrate that both governance channel and information channel play important role in the positive association. The main contribution of this article is to document the importance of information channel.*

*I enjoyed reading this paper. CSR is a trendy topic, but its economic mechanism is not well studied. I also appreciate various tests authors did to support their hypotheses. However, I have some concerns on economic mechanism and the execution of the tests.*

**Response:** Thank you very much for recognizing the revisions we've made to this before, and your comment is invaluable.

*Major concerns:*

*1) The authors could expand on the theoretical mechanisms linking analyst coverage to CSR. In particular, how does information asymmetry moderate the relationship between coverage and CSR engagement? As authors explained, the key economic mechanism, information function, refers to ‘reliable degree of external financial information’; if firms are covered by more analyst teams, investors will pay more attention to firms’ CSR level. Does it necessarily lead to monotonical increase/decrease in CSR? Some specific examples of how reduced information asymmetry impacts CSR spending would help readers better understand the posited mechanisms. Currently, the discussion focuses more on the link between asymmetry and stock prices. Clarifying the CSR mechanism specifically would make an important contribution.*

**Response:** Thanks a lot for your warmhearted instruction. Following your comments, we substantively revise the manuscript. For example:

In “Theoretical background and hypotheses” section, “First, analyst coverage could promote CSR by relieving agency problem and averting managers’ opportunistic behaviors. For routine tasks, analysts constantly monitor managers by analyzing, processing, and transmitting message about firm performance and probing into business strategies [24]. Despite uncertainty around CSR, external concerns from analyst could superintend the managers’ behavior and propel them to spend time, effort, and allocate resources to fulfill their duty to construct a better social community beyond the official legal obligations. The reduction in CSR investment, once observed by analysts, could result in considerable negative outcome of firm market value. To minimize the possibility of getting adverse feedbacks, managers would be less aggressive in reducing CSR investment and make earnest endeavors to achieve better CSR performance toward which analysts have favorable attitudes.

Second, the attention from analysts could ease the information asymmetry problem between firms and related parties. Due to the multidimensional nature of CSR, it is complex for general investors to estimate its real and intrinsic value. The valuation for firms will be discounted with incompletely conveyed CSR information [25]. Analysts could recognize the potential of CSR and factor it into investment recommendations [6]. Stock recommendations from financial analysts which represent a supposedly unbiased third party are valued by investors, especially in the developing country-level external governance environment [26]. While analysts not only pay attention to accounting fundamentals but also to other relevant professional and private information in poor information environments, analyst coverage is more influential in boosting CSR by exhibiting accurate assessment of social investment [15]. CSR is the key component of organizational sustainability, and CSR-related policies have become critical non-financial information for analysts when they prepare earnings forecasts. More analyst coverage could reduce information asymmetry and help to reasonably value corporate CSR. Considering the significant impact of analysts on the valuation of investors [26], managers will be pushed to take account of CSR when the related firms are followed and covered by analysts.”

In Discussion section, we highlight:

*“*This research enriches the prior literature by disclosing the different roles of financial analyst on CSR, by the governance channel and the information channel. On the one side, analyst coverage and firms’ inside governance mechanism complements with each other in the field of CSR. The impact of financial analyst on CSR is weaker in an inferior inside governance condition. On the other side, analyst coverage substitutes with information condition in the aspect of impacting CSR. The positive role of analyst coverage on CSR is more obvious in the worse information condition.”

“For example, the separation of CEO and chair and the participation of institutional investors are encouraged, and stock-based compensation to managers or directors needs care. The worse information situations demand analyst coverage to shape firms socially benefit behaviors more. For example, it shows that analyst coverage makes a deeper effect on CSR, in the worse information condition, specifically higher real earnings management and less accounting conservatism condition. All in all, financial analysts play different role with governance and information context. Analyst coverage complements with governance system, while substitutes for information channels to make a difference in corporate social friend activities.”

*2) The monitoring and information channels seem potentially overlapping - are they mutually exclusive? Given that gauging the information channel is a key contribution, it would be helpful for the authors to further distinguish it from the monitoring channel and quantify each one's relative contribution to the empirical results. For instance, the moderating effects of earnings news and accounting conservatism are intended to provide evidence of the information channel, but may also reflect monitoring. Discussing the degree to which the findings uniquely support the information channel would further strengthen the paper's contribution.*

**Response:** Thanks a lot for your warmhearted instruction. Following your comments, we revised the title: How does analyst coverage influence corporate social responsibility (CSR)? The governance- and information-based mechanisms

Then, we revised the Introduction section:

“In fact, investing in CSR activities is dependent on motivations of insiders such as the CEO and the board. Their evaluations on the information from analysts towards CSR are often affected by their power or discretionary decision-making. Thus, CEO duality, institutional ownership, or executive ownership could exert influence on the driving forces of analysts on corporate CSR. The concentration of the power makes CEO has the negotiating advantage among the parties and be less likely to cater to the expectations of stakeholders. Compared to the retail investors, institutional investors not only pay greater attention to various stakeholders (e.g. analysts), but also express serious concerns about societal expectations (e.g. CSR) in the oversight of managerial decision-making processes. Like CEO duality, concentrated executive ownership enlarges the power and influence of manager and could lead to the similar outcome of dual CEOs.”

In Discussion section, we highlight:

*“*This research enriches the prior literature by disclosing the different roles of financial analyst on CSR, by the governance channel and the information channel. On the one side, analyst coverage and firms’ inside governance mechanism complements with each other in the field of CSR. The impact of financial analyst on CSR is weaker in an inferior inside governance condition. On the other side, analyst coverage substitutes with information condition in the aspect of impacting CSR. The positive role of analyst coverage on CSR is more obvious in the worse information condition.”

“For example, the separation of CEO and chair and the participation of institutional investors are encouraged, and stock-based compensation to managers or directors needs care. The worse information situations demand analyst coverage to shape firms socially benefit behaviors more. For example, it shows that analyst coverage makes a deeper effect on CSR, in the worse information condition, specifically higher real earnings management and less accounting conservatism condition. All in all, financial analysts play different role with governance and information context. Analyst coverage complements with governance system, while substitutes for information channels to make a difference in corporate social friend activities.”

*3) As authors mentioned, the result could be driven by reverse causality. Authors employ 2SLS to address endogeneity, however, there are several additional tests that could be done before authors claim causality. For example, i) industry*year fixed effect in case there is any shift in the distribution of CSR ii) matching tests given endogeneity of analyst coverage (like PSM).*

**Response:** Thanks a lot for your warmhearted instruction. We adjust this part, as follow. First we added Regression Models. Then, for a specific firm, its behavior can hardly be the casual factor of its external institutions. While brokerage institution size is an external institutional factor and unlikely impossible linkage to CSR of the firms that the analysts incline to follow and coverage, hence, the change of analyst coverage driven by the difference of brokerage institutions could strength the causality, because it helps to mitigate the endogeneity concern. Moreover, prior literature in the vein of examining the relationship between financial analyst and innovation (He and Tian, 2013) choose the same instrumental variable. The identical measurement of the same variable, specifically analyst coverage, makes our paper be more consistent with the system of the whole literature. “Referring to the former results, an interesting phenomenon is observed that the magnitudes of the estimated coefficients of the 2SLS are fairly larger than the OLS. Accordingly, we can get that the coefficients of regressions are downwardly biased resulting by the problem of endogeneity. This conclusion implies that certain factors, which can both induce the firms less socially responsible and reduce the attention of analysts, are ignored. By mitigating the endogeneity problem of omitting variable, the effect of analysts on corporate socially benefit behaviors gets more positive. Therefore, we can get that the causal relationship between analysts and CSR are probably exist, by applying the 2SLS regression.”

Due to the time limitation, matching tests given endogeneity of analyst coverage (like PSM) will be conducted in the future.

*Minor issues:*

*Cross reference: Su (2019) is not cited in reference.*

**Response:** Thanks a lot for your warmhearted instruction. We added the Su (2019) in reference.

Su, K. (2019). Does religion benefit corporate social responsibility (CSR)? Evidence from China. Corporate Social Responsibility and Environmental Management, 26(6), 1206-1221.

We also new citations of up-to-date and high-quality papers on the subjects covered in manuscript. Meanwhile, we carefully format the manuscript following the guidance of the journal, such as the headers, references, and so on.

**Thanks again for your precious time, constructive comments, and valuable suggestions. We sincerely appreciate it.**

*Reviewer #2: How does analyst coverage influence corporate social responsibility (CSR)? The information-based mechanism*

*This study empirically examines whether and how analyst coverage drives CSR under different governance characteristics and information characteristics of firms. The results first show that analyst coverage positively relates to firms’ CSR. In addition, the results find that CEO duality, Institutional ownership, executive ownership, real earnings management, and accounting conservatism moderate the relationship between analyst coverage and CSR. This study did a lot of testing and it is easy to understand.*

**Response:** Thank you very much for recognizing the revisions we've made to this before, and your comment is invaluable.

*However, I have some concerns about the contribution, hypothesis development, and writing used in the study. I elaborate on my comments below.*

*Major Concerns:*

*1. Adhikari (2016) find that firms with greater analyst coverage tend to be less socially responsible, but this paper finds that the relationship between analyst coverage and CSR was positive. This research needs to explain the reasons for the difference.*

**Response:** Thanks a lot for your warmhearted instruction. Following your comments, we substantively revise the theoretical logic.

In “Theoretical background and hypotheses” section, “First, analyst coverage could promote CSR by relieving agency problem and averting managers’ opportunistic behaviors. For routine tasks, analysts constantly monitor managers by analyzing, processing, and transmitting message about firm performance and probing into business strategies [24]. Despite uncertainty around CSR, external concerns from analyst could superintend the managers’ behavior and propel them to spend time, effort, and allocate resources to fulfill their duty to construct a better social community beyond the official legal obligations. The reduction in CSR investment, once observed by analysts, could result in considerable negative outcome of firm market value. To minimize the possibility of getting adverse feedbacks, managers would be less aggressive in reducing CSR investment and make earnest endeavors to achieve better CSR performance toward which analysts have favorable attitudes.

Second, the attention from analysts could ease the information asymmetry problem between firms and related parties. Due to the multidimensional nature of CSR, it is complex for general investors to estimate its real and intrinsic value. The valuation for firms will be discounted with incompletely conveyed CSR information [25]. Analysts could recognize the potential of CSR and factor it into investment recommendations [6]. Stock recommendations from financial analysts which represent a supposedly unbiased third party are valued by investors, especially in the developing country-level external governance environment [26]. While analysts not only pay attention to accounting fundamentals but also to other relevant professional and private information in poor information environments, analyst coverage is more influential in boosting CSR by exhibiting accurate assessment of social investment [15]. CSR is the key component of organizational sustainability, and CSR-related policies have become critical non-financial information for analysts when they prepare earnings forecasts. More analyst coverage could reduce information asymmetry and help to reasonably value corporate CSR. Considering the significant impact of analysts on the valuation of investors [26], managers will be pushed to take account of CSR when the related firms are followed and covered by analysts.”

*2. The author uses " The information-based mechanism " as the subtitle of the article. However, in the hypotheses part, the author did not test how can analysts reduce information asymmetry and thus affect CSR. Instead, the author states that poor corporate governance and low financial information quality increase the space for analysts to influence CSR. This description proves that corporate governance can complement the effect of analyst supervision (from the perspective of supervision rather than from the perspective of information asymmetry).*

**Response:** Thanks a lot for your warmhearted instruction. Following your comments, we revised the title: How does analyst coverage influence corporate social responsibility (CSR)? The governance- and information-based mechanisms

*The above problems lead to two extended problems:*

*(1) In the introduction part, the author skipped the description of* *CEO duality, Institutional ownership, and executive ownership. Instead, it describes real earnings management and accounting conservatism directly after describing the impact of analysts on CSR (see page 5: The information heterogeneity, such as various accounting information characters, such as real earnings management and accounting conservatism…).*

**Response:** Thanks a lot for your warmhearted instruction. Following your comments, we revised the Introduction section:

“In fact, investing in CSR activities is dependent on motivations of insiders such as the CEO and the board. Their evaluations on the information from analysts towards CSR are often affected by their power or discretionary decision-making. Thus, CEO duality, institutional ownership, or executive ownership could exert influence on the driving forces of analysts on corporate CSR. The concentration of the power makes CEO has the negotiating advantage among the parties and be less likely to cater to the expectations of stakeholders. Compared to the retail investors, institutional investors not only pay greater attention to various stakeholders (e.g. analysts), but also express serious concerns about societal expectations (e.g. CSR) in the oversight of managerial decision-making processes. Like CEO duality, concentrated executive ownership enlarges the power and influence of manager and could lead to the similar outcome of dual CEOs.”

*(2) H3 does not clarify why institutional ownership positively moderates the relationship between analyst coverage and CSR.*

Thanks a lot for your warmhearted instruction. Following your comments, we revised the H3 section:

**Response:** “Risk is a significant factor considered by the institutional investors. Institutional investors are reluctant to pick and invest in the risky stocks, ceteris paribus [30](Graves and Waddock, 1994). Usually, firms with strong CSR have less systematic risk and greater growth opportunity [31, 32](Doh et al., 2010). As a result, institutional investors favor corporate engagements in CSR.

As important shareholders, institutional owners are more concerned about and actively involved in organizational strategic decisions than non-institutional stockholders. The rapid growth of stakeholder demands, fund flow benefits, and risk reduction are the reasons that institutional investors prioritize firms with social benefit and sustainable goals. Accordingly, institutional investors are inclined to seek out higher-CSR-performing firms and enlarge the related share of their portfolios [33](Chen et al., 2020). Compared to the scatter investors, the institutional investors have the ability to hold the stock for a longer time, and hence are probably more care about the long-term activities, such as CSR [34]. The CSR of firms, with more institutional investors and more followed analysts, can meet the benefits of the two parties, but not the financial analysts alone. Therefore, the firms have more motivation to engage in CSR when face high institutional ownership and financial coverage. Most institutional investors are long-term orientated with greater tolerance for decreasing of short-term profits [35]. The more tolerance investors have, the less pressure analyst average conducts, and the more contemplations and motivations managers will be inspired to take the opportunities and bear the uncertainty of CSR.”

*I suggest that the author clarify what governance characteristics and information characteristics actually represent and write them out clearly in hypotheses development. Do not let the reader feel that it is supervision, rather than information. Or the author can directly focus on supervision.*

**Response:** Thanks a lot for your warmhearted instruction. We have incorporated the suggested revisions into this refined manuscript to the best of our ability. We have benefited greatly from these insightful suggested revisions.

*Minor concerns:*

1. *The statistics of the real earnings management and accounting conservatism variables should be shown in Tables 1 and 2.*

**Response:** Thanks a lot for your warmhearted instruction. We have added the statistics of the real earnings management and accounting conservatism variables in Tables 1 and 2.

*2. The authors need to explain why this research does not use the accrual earnings as Information characteristics, or supplement the result.*

**Response:**

*3. The article has spelling or punctuation errors that need to be corrected carefully.*

**Response:** Thanks a lot for your warmhearted instruction. We carefully examine and proof the language. We also include new citations of up-to-date and high-quality papers on the subjects covered in manuscript. Meanwhile, we carefully format the manuscript following the guidance of the journal, such as the headers, references, and so on.

**Thanks again for your precious time, constructive comments, and valuable suggestions. We sincerely appreciate it.**
