# Supplementary material for: How does analyst coverage influence corporate social responsibility (CSR)? The governance- and information-based perspectives
Source: PLoS One. 2024 Apr 29;19(4):e0302165. doi: 10.1371/journal.pone.0302165 (PMC11057776; doi:10.1371/journal.pone.0302165)
Supplement: Attachment — Submitted filename: Response letter.docx [file pone.0302165.s003.docx]

**Response Letter**

Dear Editor and Reviewers,

It is with excitement that we resubmit to you a revised version of manuscript “**How does analyst coverage influence corporate social responsibility (CSR)? The governance- and information-based perspectives (PONE-D-23-17772)**” for the ***Plos One***. Many thanks to all the editors for their valuable time, constructive comments, and valuable suggestions. Based on the review comments, we completed the revision of the manuscript with the full cooperation of the author team and resubmitted the new version before the deadline. We thank you for your time and details provided. We have incorporated the suggested revisions into this refined manuscript to the best of our ability. We have benefited greatly from these insightful suggested revisions. We look forward to working with you to bring the manuscript closer to publication in ***Plos One***.

Based on the editor and reviewers’ comments and suggestions, we have carefully revised the manuscript. The following is a detailed response to the editor and reviewers’ comments.

Reviewer #1: Thank you for revising the manuscript based on previous review. I carefully examine the revised version. While some improvements have been made, there are still some issues need to be addressed before the manuscript can be considered for publication.

About comment 1:

I appreciate authors’ effort in providing examples of positive association between analyst coverage and CSR. However, authors need to further explain the underlying mechanism of the positive association. Simply saying CSR is an important information source does not explain why higher analyst scrutiny incentivize firms to increase CSR engagement. For example, would firms significantly increase CSR investment just to send a noisy signal to the market, even if they think CSR is informative? The authors need to develop a more cogent argument for the positive sign.

**Response:** Thanks a lot for your warmhearted instruction. Following your comments, we revised the argument for the hypotheses (see the revised manuscript with track changes) and hope to meet your high expectations.

About comment 2:

I recognize authors’ adjustment on the title and in hypothesis section. I am satisfied with the adjustment.

**Response:** Thank you very much for recognizing the revisions we've made to this before, and your comment is invaluable.

About comment 3:

I appreciate authors’ effort on constructing instrument variables like brokerage institution. I am satisfied with the revised robustness section.

**Response:** Thank you very much for recognizing the revisions we've made to this before, and your comment is invaluable.

In summary, while progress has been made, further revisions are still needed to address my previous concerns. I hope these comments are helpful. Please let me know if you have any further concerns.

**Thanks again for your precious time, constructive comments, and valuable suggestions. We sincerely appreciate it.**

Reviewer #2: How does analyst coverage influence corporate social responsibility (CSR)? The governance- and information-based mechanism

According to the comments of the reviewers, the authors have greatly revised the article, and it can be seen that the quality of the article has been significantly improved. However, I think there are still some contents in the article that need to be modified. Here are my comments:

1. In the title, using the word "mechanism" to represent "moderators" is considered unprofessional. Few top journals use this expression, and the authors should avoid the use of "Chinglish."

**Response:** Thanks a lot for your warmhearted instruction. Following your comments, we revised the title: “How does analyst coverage influence corporate social responsibility (CSR)? The governance- and information-based perspectives”.

2. The authors mentioned in introduction that “few studies have disclosed the impact of external analysts' attention on a firm's CSR and its underlying information mechanisms”, but I think this description is inappropriate, there is already a lot of relevant literature.

**Response:** Thanks a lot for your warmhearted instruction. Following your comments, we revised the expression: “Recently, a strand of attention has been paid to disclosed the impact of external analysts’ attention on a firm’s CSR and its underlying mechanisms [13].”

3.In introduction, the authors list some of the literature, then mentioned that “However, as the theorical analysis implied in the prior literature, analyst coverage may work on CSR through the governance- and information-based mechanism”, however, it is not obvious to me that this conclusion can be drawn from the preceding literature. From the sentence “In fact, investing in CSR activities is dependent on motivations of insiders such as the CEO and the board. Their evaluations on the information from analysts towards CSR are often affected by their power or discretionary decision-making.", I also cannot conclude why CEO duality, institutional ownership, or executive ownership could exert influence on the driving forces of analysts on corporate CSR. The authors need to pay attention to the logic when using connectionists such as "in fact" and "thus".

**Response:** Thanks a lot for your warmhearted instruction. Following your comments, we revised the expression and adjusted the logic: “These studies offer evidence that financial analysts could exert influence on CSR through their governance or information related functions. In practical, investing in CSR activities is dependent on motivations of insiders such as the CEO and the board. Their evaluations on the information from analysts towards CSR are often affected by their power or discretionary decision-making. The concentration of the power makes CEO has the negotiating advantage among the parties and be less likely to cater to the expectations of stakeholders. Compared to the retail investors, institutional investors not only pay greater attention to various stakeholders (e.g. analysts), but also express serious concerns about societal expectations (e.g. CSR) in the oversight of managerial decision-making processes. Like CEO duality, concentrated executive ownership enlarges the power and influence of manager and could lead to the similar outcome of dual CEOs. Thus, CEO duality, institutional ownership, or executive ownership might be the driving forces of analysts on corporate CSR to a certain extent.”

4. Before the sentence “As a result, the comparisons of the moderate effect of corporate governance and external financial information are necessary.” The author should explain the reasons for using financial information as moderator in more detail.

**Response:** Thanks a lot for your warmhearted instruction. Following your comments, we added explanations for supplementing the moderators:

“Additionally, the information heterogeneity, such as various accounting information characters (e.g. real earnings management and accounting conservatism), may result in a different impact of analyst coverage on CSR, which is scarcely empirically examined in the prior studies. Meanwhile, both real earnings management and accounting conservatism reflect the reliable degree of external financial information [19, 20]. Real earnings management emphasizes whether the accounting information is manipulated through real activities, such as adjusting production and producing cost. Accounting conservatism positively relates to the timeliness of confirming bad news, while negatively collated to good news. The process of considering financial analysts’ opinions into CSR behaviors cannot ignore the necessary conditions which comprise reactions from the capital market. It is necessary to specially disclose the role of external financial information in corporate strategies towards CSR investments. While the characteristics of external financial information is significant different from corporate governance system, the comparisons of the effects of corporate governance and external financial information are also relevant.”

5.In Hypothesis 3, the authors do not well describe why institutional ownership affect the relationship between analysts and CSR, but spend a lot of words on how institutional investors affect CSR, which does not have much relevance to the hypothesis. At the same time, the author mentioned that “Therefore, the firms have more motivation to engage in CSR when face high institutional ownership and financial coverage.” would make readers question the causal relationship between analysts and CSR, which is not in line with the title of this paper” How does analyst coverage influence corporate social responsibility”. Similarly, in Hypothesis 4-6, authors need to add the description of why executive ownership/earnings management/conservatism affects the relationship between analysts and CSR (Instead of just describing why executive ownership/earnings management/conservatism affects CSR).

**Response:** Thanks a lot for your warmhearted instruction. Following your comments, we revised the argument for the hypotheses. For example:

“Being a strong symbol of prosocial responsibility, CSR naturally is regarded as an important indicator for investment decisions. Institutional investors often have a shared understanding of the value of CSR with analysts for the following firms.” in Hypothesis 3.

“Firms with high executive ownership often have less motivations to engage in CSR although analysts are encouraging corporate investments in CSR. For example, firms cut off charitable donation after an increase in executive ownership [37]. Thus, a high level of executive ownership will lead to the constraint of analysts and pay less attention to analysts’ opinions, such as prioritizing CSR.” in Hypothesis 4.

“In other words, financial analysts play a more profound role on CSR in firms with larger degree of real earnings management. Firms naturally have more motivations to engage in CSR to go along with analysts when they have special preference to better CSR performance.” in Hypothesis 5.

“In other words, accounting conservatism may be substitute with financial analysts by regulating the agent parties. The value guidance from analysts is likely to be given a smaller weight in this event. Although analysts have special preference to CSR, managers often choose to turn a blind eye to these opinions.” in Hypothesis 6.

5. In Data and samples, the authors need to describe why only manufacturing firms was selected.

**Response:** Thanks a lot for your comments. Manufacturing firms have been chosen because they are the mainspring of economy development and the main sources of air, land, and water pollution in China. The research on their CSR is quite relevant for the sustainability of the country.

6. The authors need to explain why this research does not use the accrual earnings as Information characteristics, or supplement the result.

**Response:** Thanks a lot for your comments. In this study, we did not use accrual earnings as information characteristics because we think real earnings management is more difficult to detect than accrual-based earnings management and is constrained by analyst following (Enomoto et al., 2015). The influence of analyst coverage on CSR would be more sensitive by employing real earnings management as the information characteristics.

Enomoto, M., Kimura, F., & Yamaguchi, T. (2015). Accrual-based and real earnings management: An international comparison for investor protection. Journal of Contemporary Accounting & Economics, 11(3), 183-198.

**Thanks again for your precious time, constructive comments, and valuable suggestions. We sincerely appreciate it.**
